# Carbohydrate quality indices and colorectal cancer risk: a case-control study

**DOI:** 10.1186/s12885-023-10786-6

**Published:** 2023-04-17

**Authors:** Masoud Amini Kahrizsangi, Zohreh Ebrahimi, Zainab Shateri, Fatemeh Mansouri, Ali Zangene, Milad Rajabzadeh-Dehkordi, Mehran Nouri, Bahram Rashidkhani

**Affiliations:** 1 Department of Community Nutrition, School of Nutrition and Food Sciences, Isfahan University of Medical Sciences, Isfahan, Iran; 2 Department of Nutrition, School of Public Health, Iran University of Medical Sciences, Tehran, Iran; 3 Student Research Committee, Ahvaz Jundishapur University of Medical Sciences, Ahvaz, Iran; 4 Department of Clinical Nutrition, School of Nutrition and Food Sciences, Shiraz University of Medical Sciences, Shiraz, Iran; 5 Student Research Committee, Shiraz University of Medical Sciences, Shiraz, Iran; 6 Department of Community Nutrition, School of Nutrition and Food Sciences, Shiraz University of Medical Sciences, Shiraz, Iran; 7 Health Policy Research Center, Institute of Health, Shiraz University of Medical Sciences, Shiraz, Iran; 8 Department of Community Nutrition, Faculty of Nutrition and Food Technology, National Nutrition and Food Technology Research Institute, Shahid Beheshti University of Medical Sciences, Tehran, Iran

**Keywords:** Glycemic load, Insulin load, Glycemic index, Insulin index, Low-carbohydrate diet score, Carbohydrate quality index, Colorectal cancer

## Abstract

**Background:**

Colorectal cancer (CRC) is the fourth and third most common cancer in Iran and the world, respectively. Carbohydrates can lead to the proliferation of cancer cells, including CRC. The current study aimed to investigate the association between glycemic load (GL), insulin load (IL), glycemic index (GI), insulin index (II), low-carbohydrate diet score (LCDS), and carbohydrate quality index (CQI) with CRC odds.

**Methods:**

The present case-control study was performed on 71 CRC cases and 142 controls in the Hospital Cancer Organization and three general hospitals in Tehran, Iran. We calculated the dietary GI, GL, IL, II, CQI, and LCDS by a validated food frequency questionnaire.

**Results:**

The results indicated that people who were in the highest tertile of the GI had higher odds of CRC compared to the lower tertile (in the adjusted model: odds ratio (OR) = 3.89; 95% confidence interval (CI): 1.71–8.84). On the contrary, people who were in the highest tertile of the CQI and LCDS had significantly lower odds of CRC compared to the lower tertile (in the adjusted model: tertile (T) _2_-OR = 0.24; 95% CI: 0.11–0.53 and T_3_-OR = 0.15; 95% CI: 0.06–0.39 for CQI and T_2_-OR = 0.33; 95% CI: 0.13–0.79 and T_3_-OR = 0.28; 95% CI: 0.10–0.82 for LCDS). Also, IL was positively associated with the odds of CRC after adjusting for confounding factors (T_2_-OR = 2.46; CI: 1.08–5.61 and T_3_- OR = 2.80; 95% CI: 1.07–7.31). Regarding the GL, only individuals who were in the second tertile had significantly higher odds of CRC compared to the first tertile (OR = 2.42; CI: 1.07–5.47).

**Conclusion:**

According to the findings, it is recommended to use a diet with high-quality carbohydrates and low GI and GL to minimize the odds of developing CRC. People should also be encouraged to have a balanced carbohydrate intake.

## Introduction

Colorectal cancer (CRC) is the fourth and third most common cancer in Iran and the world, respectively [1–3]. The incidence and mortality rates of CRC have been increasing in recent years [4], so that in 2020, CRC accounted for 1.14 million new cases worldwide [5]. Various factors (modifiable and non-modifiable risk factors) affect CRC development [6]. Epidemiological studies have shown a relationship between diets and CRC risk [7–9]. Some dietary factors, including carbohydrates, lead to the proliferation of cancer cells, including CRC, through alternations in insulin levels and circulating glucose, impaired glucose metabolism, insulin resistance, and hyperinsulinemia [10]. The ability of carbohydrates to affect blood glucose and insulin concentrations differs substantially and depends on the diet’s amount, composition, and quality [11, 12]. Glycemic load (GL), glycemic index (GI), carbohydrate quality index (CQI), and low-carbohydrate diet score (LCDS) are used to assess the quantity and quality of carbohydrates in foods [13, 14]. The GI indicates how food’s carbohydrate content influences blood glucose levels [15]. Also, GL offers the impact of all dietary carbohydrates on glucose after a meal [11]. However, a single component cannot be a suitable criterion for assessing the quality of carbohydrates, so the CQI was introduced as an indicator of dietary carbohydrate quality that consists of the GI and dietary fiber intakes, whole grains, and solid or liquid carbohydrates [16]. Overall, all data collectively support a central role of glucose metabolism in carcinogenesis and lead to significant interest in LCDS as a practical dietary approach for cancer prevention [17].

It has been shown that when assessing the insulin response, carbohydrates are not the only stimulus for its release, so the insulin load (IL) and dietary insulin index (II) [18] were presented [19]. II indicates the insulin response after a meal, including protein, fat, and carbohydrates, compared to an isoenergetic portion of a reference meal (white bread or glucose). IL is also computed by multiplying each food’s II by its consumption frequency and energy content [20].

Previous studies have investigated the relationship between GL, GI, IL, and II with CRC risk, and the potential relationship between glucose metabolism and cancer is still debated [21–24]. Furthermore, despite the important role of CQI in assessing the quality of carbohydrates, studies have not yet evaluated the association between CQI and LCDS with CRC odds. To our knowledge, previous research has not yet simultaneously demonstrated the association of overall quality and quantity of carbohydrate intake on the odds of CRC. In conclusion, the findings of this research contribute to understanding the potential relationship between total carbohydrate intakes and CRC odds. Therefore, the current study investigated the association between CQI, LCDS, and other indices (GL, GI, IL, and II) and the odds of CRC.

## Methods

### Study population

**Fig. 1:**
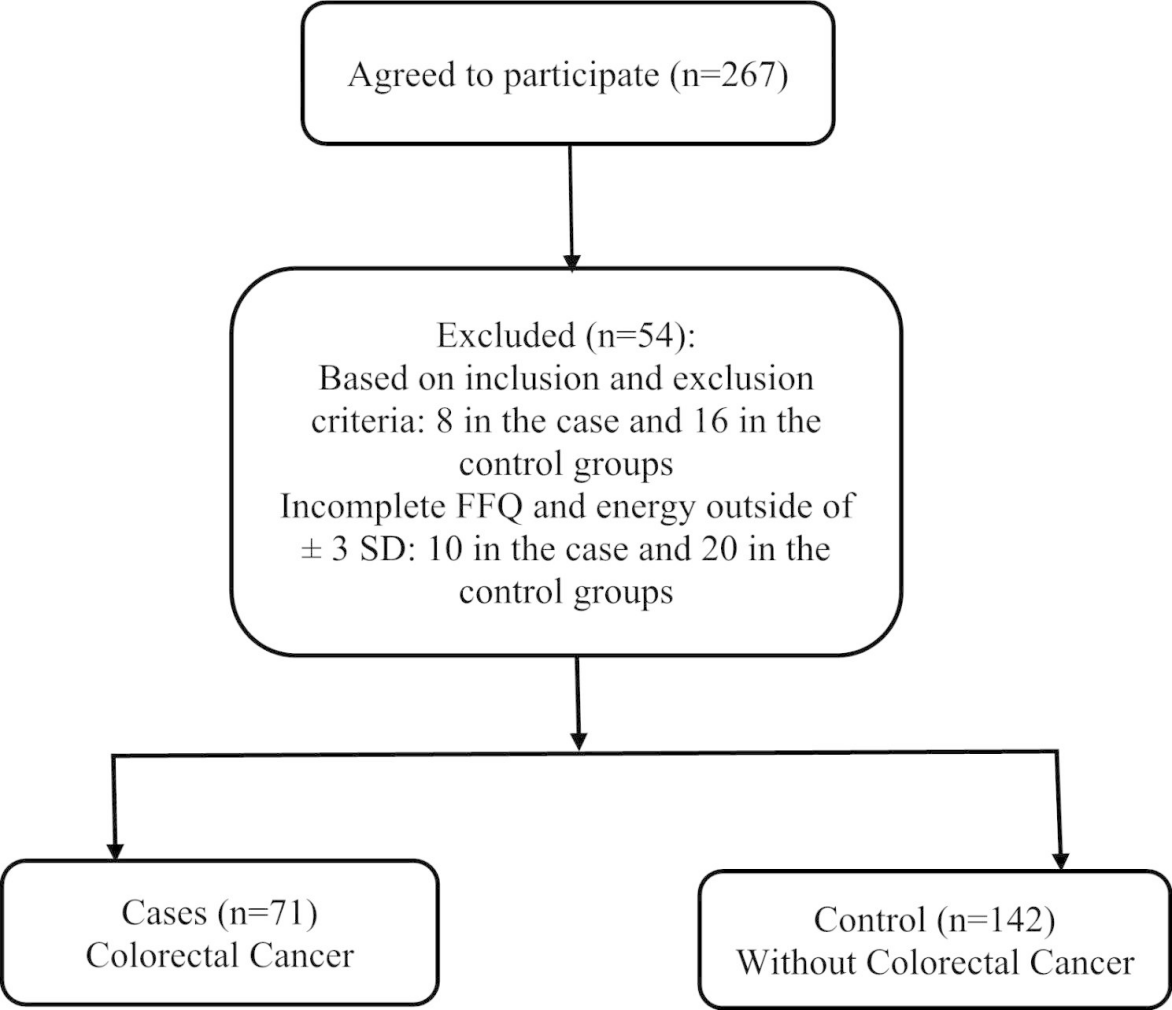
Flow chart of the study

This study was conducted in hospital and in 19 CRC surgery departments of Imam Khomeini Hospital Cancer Organization and three general hospitals in Tehran, Iran (convenient sampling from September 2008 to January 2010).The sample size was calculated based on the previous study [25] considering odds ratio (OR) = 0.45, α = 0.05, β = 0.2. In the case group, people (40–75 years old) who were newly diagnosed with CRC by pathological assessment ≤ 6 months before the interview and had no previous cancer diagnosis in other organs or a history of adenomatous polyposis were included. The control group’s characteristics included a random selection from the same hospitals and hospitalization for acute and non-neoplastic circumstances during the same time, without chronic diseases related to diet. The most reasons for hospitalization included fractures and sprains, osteoarticular disorders, and disk disorders. Also, the case and control groups were matched according to age and sex. Initially, 89 people were selected for the case group, and 178 people were selected for the control group, who were screened based on the inclusion and exclusion criteria (24 people were excluded (16 controls and 8 cases)). Moreover, 30 other patients (20 controls and 10 cases) were excluded because of incomplete food questionnaire (more than 40% of food items not answered) and total energy intake (outside of mean ± three standard deviations (SDs)) and an unfinished food frequency questionnaire (FFQ) (Fig. 1). The statistical analysis was performed on 142 controls and 71 cases.

### Dietary assessment

A 168-item semi-quantitative FFQ utilized for this study, and the reliability and validity of questionnaire were assessed before [26]. Data collected from FFQ were changed to daily intake, and the food intakes were converted to grams [27]. Finally, we applied Nutritionist IV (N IV) (version 7.0; N-Squared Computing, Salem, OR, USA) to calculate energy and nutrient intake [28].

### Diet quality scores

GI was computed by this formula: (GI × available carbohydrate)/total available carbohydrate. Available carbohydrate means carbohydrate minus fiber [29]. Food carbohydrate and fiber contents were taken from the United States Department of Agriculture food-composition table. From 85 foods, the Iranian GI table covered six foods [29]. For 62 foods, international tables were used [29], and similar foods were estimated for the other food items because the amounts of GI were not available, like some desserts or traditional sweets such as Gaz. The GIs of mixed meals were determined by the GIs of each food component of the meal [29]. Then, GL was calculated by this formula: (total GI × total available carbohydrate/100).

II shows the increasing insulin zone under the two hours curve in response to the use of 1000 kilojoules (kj) [30] of the trial food divided by the zone under the curve of the utilization of 1000 kj of the reference food. The II of foods was taken from a previously published study [31]. The II of identical food was used for food items in our research that were not reachable in the former studies. For example, for dates, raisins were used. Also, to assess dietary IL, the IL of every food was computed by this formula: IL = II of food × energy content of that food [32]. Then, the IL of every food was summed, and IL was obtained for every participant. Finally, the dietary intake for every participant was calculated: IL/ total energy intake.

Also, to evaluate CQI, four criteria were considered: first, the carbohydrate ratio of whole grains to those of total grains; second, the GI; third, the proportion of solid carbohydrates to total carbohydrates; fourth, the total fiber of diet. The total score was between 4 and 20. A higher score means better quality of carbohydrates [33, 34].

Moreover, to determine LCDS, the individuals were divided into 11 categories for intake of carbohydrates, vegetable protein, refined grains, n3/n6 polyunsaturated fatty acid (PUFA), monounsaturated fatty acid (MUFA), fiber, and GL [35]. For MUFA, PUFA, fiber, and vegetable protein, the highest category got 10 points, and the lowest category got 0 points. The scoring method was reversed for GL, refined grains, and carbohydrates (the lowest carbohydrate got 10 points, and the highest carbohydrate got 0 points). For the overall diet score, the points of every item were summed, and the range was between 0 and 70. The 0 score means the lowest intake of fat and protein and the highest intake of carbohydrates, and the 70 score means the maximum fat and protein intake and the minimum carbohydrate intake. Finally, a higher score indicates more adherence to a LCD.

### Socio-demographic and anthropometric assessments

Information (physical activity, dietary intake, CRC history, medication use, and socio-demographic characteristics) was collected by trained interviewers. The anthropometric indices were determined. Weight (with a precision of 0.1 kg) and height (determined by SECA body meter with a precision of 0.1) were measured. By the following formula, body mass index (BMI) was calculated: weight (kg) / height (m)^2^ [36]. International Physical Activity Questionnaire (IPAQ) was used to determine physical activity [37]. For the case patients, the activities of the year before the diagnosis of CRC and for the control subjects, the activities of the year before the interview were considered.

### Statistical analysis

For statistical analysis, SPSS (version 26.0, SPSS Inc. Chicago IL, USA) was applied. The Kolmogorov-Smirnov test was used to check the normality of the data. Means (SD) or median (interquartile range (IQR)) and frequencies (percentage) were used for continuous and categorical variables, respectively. The chi-square test was applied for categorical variables, and the Mann-Whitney U test and independent samples T-test were used for continuous variables. To assess the correlation between CRC and indices, crude and adjusted model logistic regression were used. The role of BMI, energy intake, physical activity, smoking, history of CRC, and taking ibuprofen, aspirin, acetaminophen, and vitamin/mineral supplement was adjusted in the model. P-value < 0.05 was considered as the level of statistical significance.

## Results

The baseline characteristics of the study population are presented in Table 1. The mean and SD of the age of the participants in the case and control group were 58.2 ± 10.4 and 57.7 ± 10.4, respectively (P = 0.746). Also, the BMI of the case group was 27.6 ± 4.2 and the control group one was 26.6 ± 4.2 (P = 0.362). There were significant differences in the history of CRC (P = 0.017), taking aspirin (P = 0.016), acetaminophen (P = 0.004), and vitamin/mineral supplementation (P = 0.015) between the two groups. Regarding dietary intake, the intake of protein (P = 0.048) and fiber (P < 0.001) was lower in the case group than in the control group. In contrast, the fat intake (P < 0.001) was significantly higher in the case group than the control group. GI was greater in the case group than in the control group (P = 0.001). However, the total LCDS was higher in the control group than in the case group (P < 0.001). There were no significant differences in II and CQI between the case and control groups (P > 0.05).

**Table 1:** The basic characteristic of the control (n = 71) and case groups (n = 142)

Variables	Cases (71)	Controls (142)	P-value
Age (year) ^1^	58.2 ± 10.4	57.7 ± 10.4	0.746
Energy (kcal/day) ^1^	2262.3 ± 450.1	2255.2 ± 341.2	0.908
Carbohydrate (g/day) ^1^	347.5 ± 89.6	354.8 ± 71.8	0.552
Protein (g/day) ^1^	79.1 ± 17.2	83.8 ± 14.3	**0.048**
Fat (g/day) ^1^	65.8 ± 8.1	60.5 ± 8.4	**<0.001**
Fiber (g/day) ^1^	18.9 ± 2.3	20.4 ± 3.1	**<0.001**
Glycemic index ^2^	63.6 (5.9)	61.7 (6.6)	**0.001**
Insulin index ^2^	44.0 (17.4)	41.7 (14.7)	0.087
CQI total score ^2^	11.0 (4.0)	13.0 (3.0)	0.177
Total LCDS^2^	34.0 (22.0)	36.0 (13.2)	**<0.001**
BMI (kg/m^2^) ^1^	27.6 ± 4.2	26.6 ± 4.2	0.362
Income (dollar) ^2^	393.0 (253.0)	402.0 (302.0)	0.206
Physical activity (MET-h/day) ^1^	36.8 ± 3.6	36.7 ± 4.8	0.932
History of CRC, % ^3^			**0.017**
Yes	7 (9.9)	3 (2.1)	
No	64 (90.1)	139 (97.9)	
Smoking, % ^3^			0.164
Never	57 (80.2)	101 (70.1)	
Former	8 (11.3)	15 (10.6)	
Current	6 (8.5)	26 (18.3)	
Ibuprofen, % ^3^			
Yes	5 (7.0)	22 (15.5)	
No	66 (93.0)	120 (84.5)	
No	70 (98.6)	128 (90.1)	
Acetaminophen, % ^3^			**0.004**
Yes	4 (5.6)	28 (19.7)	
No	67 (94.4)	114 (80.3)	
Taking vitamin and mineral supplements, % ^3^			**0.015**
Yes	8 (11.3)	35 (24.6)	
No	73 (88.7)	107 (75.4)	

MET: metabolic equivalent of task, CQI: carbohydrate quality index, LCDS: low carbohydrate diet score, BMI: body mass index, CRC: colorectal cancer

Values are mean ± SD for continuous and frequency (percentage) for categorical variables

^1^ Using independent samples T-test for normal continuous variables.

^2^ Using Mann-Whitney U test for abnormal continuous variables.

^3^ Using chi-square test for categorical variables.

Table 2 shows ORs and 95% confidence intervals (CIs) in the multivariable-adjusted and crude models across tertile of GI, GL, II, IL, CQI, and LCDS. As can be seen, in the adjusted model, the odds of CRC in the third tertile were significantly higher than the first tertile of GI (OR = 3.89; 95% CI: 1.71–8.84). Also, we found a significant association between the second tertile of GL and the odds of CRC in the adjusted model (OR = 2.42; 95% CI: 1.07–5.47). Moreover, there was a significant association between IL and CRC odds in the second and last teritles compared to the first tertile in the adjusted model (tertile (T)_2_-OR = 2.46; CI: 1.08–5.61 and T_3_-OR = 2.80; 95% CI: 1.07–7.31). In contrast, there was a negative relationship between CQI and CRC in the adjusted model (T_2_-OR = 0.24; 95% CI: 0.11–0.53 and T_3_-OR = 0.15; 95% CI: 0.06–0.39). Also, individuals in the second and third tertiles of LCDS had lower odds of CRC than the first tertile (T_2_- OR = 0.33; 95% CI: 0.13–0.79 and T_3_- OR = 0.28; 95% CI: 0.10–0.82).

**Table 2:** Crude and multivariable-adjusted OR and 95% CIs across tertile of GI, GL, II, IL, CQI, and LCDS (in 71 cases and 142 controls)

Variables	Case/Control	Crude	Adjusted Model
**GI**	71/142		
T_1_	17/54	1.00 (Reference)	1.00 (Reference)
T_2_	21/50	1.33 (0.63–2.81)	1.35 (0.59–3.04)
T_3_	33/38	**2.75 (1.34–5.65)**	**3.89 (1.71–8.84)**
P_trend_		**0.005**	**0.001**
**GL**	71/142		
T_1_	19/52	1.00 (Reference)	1.00 (Reference)
T_2_	28/43	1.78 (0.87–3.62)	**2.42 (1.07–5.47)**
T_3_	24/47	1.39 (0.68–2.87)	1.85 (0.73–4.70)
P_trend_		0.374	0.165
**II**	71/142		
T_1_	21/50	1.00 (Reference)	1.00 (Reference)
T_2_	23/48	1.14 (0.56–2.32)	1.08 (0.48–2.42)
T_3_	27/44	1.46 (0.72–2.94)	1.82 (0.80–4.13)
P_trend_		0.286	0.122
**IL**	71/142		
T_1_	18/53	1.00 (Reference)	1.00 (Reference)
T_2_	27/44	1.91 (0.92–3.95)	**2.46 (1.08–5.61)**
T_3_	26/45	1.87 (0.90–3.86)	**2.80 (1.07–7.31)**
P_trend_		0.098	**0.027**
**CQI**	71/142		
T_1_	30/32	1.00 (Reference)	1.00 (Reference)
T_2_	26/65	**0.34 (0.17–0.68)**	**0.24 (0.11–0.53)**
T_3_	15/45	**0.27 (0.12–0.59)**	**0.15 (0.06–0.39)**
P_trend_		**0.001**	**<0.001**
**LCDS**	71/142		
T_1_	30/40	1.00 (Reference)	1.00 (Reference)
T_2_	18/53	**0.45 (0.22–0.92)**	**0.33 (0.13–0.79)**
T_3_	23/49	0.62 (0.31–1.24)	**0.28 (0.10–0.82)**
P_trend_		0.173	**0.021**

GI, glycemic index; GL, glycemic load; II, insulin index; IL, insulin load; CQI, carbohydrate quality index; LCDS, low-carbohydrate diet score.

Adjusted for BMI, energy intake, physical activity, smoking, history of CRC, and taking ibuprofen, aspirin, acetaminophen, and vitamin/mineral supplement.

-These values are presented as odds ratio (95% CIs).

-Obtained from logistic regression.

## Discussion

Our research indicated a positive association between GI, GL, IL, and CRC odds. Furthermore, we found a negative association between CQI, LCDS, and CRC odds.

A compound of genetic and environmental factors, especially diet, plays a role in cancer etiology [38, 39]. The current study demonstrated a significant positive association between dietary GL and GI and CRC odds. GI and GL indicate different dimensions of consumed carbohydrates. GI provides information about the overall quality of carbohydrates in the diet. In contrast, dietary GL, which reflects the amount of carbohydrate intake, contains both the quantity and quality of carbohydrate intake in the diet [40].

A study by Choi et al. revealed that dietary GI has a positive and significant relationship with the risk of CRC [41]. Moreover, the findings from a prospective study showed that increasing consumption of a high GI diet was significantly related to an increased risk of CRC [42]. Consumption of a high glycemic diet is equal to high blood glucose levels. The amount of serum insulin increases after glucose rises. This hormone arouses cancer growth by decreasing insulin-like growth factor (IGF) binding protein, increasing the bioactivity of IGF-1, and changing the metabolism of the sex hormone [11]. Research has shown that high IGF-1 and C-peptide (indicating increased insulin rates) are related to a remarkable increase in CRC risk [43, 44].

Our findings suggested that higher IL might be associated with higher odds of CRC. IL, which has taken significant consideration in recent years, is a suitable indicator to predict the risk of chronic diseases [45, 46]. Similar to our observations, a study revealed that higher IL was associated with CRC risk [47]. In another study, it was observed that higher scores of dietary insulin were associated with a statistically significant increment in mortality after CRC diagnosis [48]. Furthermore, it has been reported that higher II is significantly related to a higher risk of recurrence and mortality in patients with colon cancer [49]. In contrast to our results, a prospective study showed that dietary IL were not associated with the risk of CRC [24].

Also, a significant negative relationship was observed between LCDS and the odds of CRC. Several studies have shown an association between LCD and some cancers [50–52]. A prospective study by Cai et al. indicated that animal-based LCD was related to a greater risk of CRC [52]. Moreover, Song et al. illustrated that vegetable-based LCD was related to lower CRC-specific mortality [53]. It has been shown that both hyperinsulinemia and hyperglycemia are associated with a poor prognosis of CRC [54–56]. These studies demonstrated the critical role of glucose metabolism in carcinogenesis and led to considerable interest in LCD as a beneficial dietary approach to help in cancer treatment [17]. In addition, as mentioned earlier, high carbohydrate intake and high GL may increase blood glucose, and as a result, insulin increases [18, 57]. Insulin has been shown to stimulate cancer cells, reduce apoptosis, and can increase carcinogenesis through IGF-1 [58]. As a result, it seems that LCD can reduce the odds of cancer by the mentioned mechanisms.

Also, the findings demonstrated an inverse association between CQI and CRC odds. Despite the important role of CQI in evaluating the quality of carbohydrates, no studies have evaluated the association between CQI and CRC odds. However, an inverse relationship has been observed between higher CQI diets and breast cancer risk [59], obesity [60], cardiovascular disease incidence [33], and all-cause mortality [61], compared to lower CQI diets. In line with our findings in the study of Sasanfar et al. it was also found that greater CQI scores were related to a lower breast cancer risk [59].

The strengths of the present study are that despite the significant role of CQI in assessing the quality of carbohydrates, studies have not yet evaluated the association between CQI and LCDS and the odds of CRC with this method. Also, we used valid and reliable questionnaires for data collection [26], which can further support the accuracy of the findings. In addition, our study has several limitations that should be noted. First, the study sample size was small. Second, the data are case-control, which prevents us from concluding the causal relationships between the variables. Third, we used similar foods for the limited number of foods that II was unavailable. Therefore, further II tests are required to support our results in the present study.

## Conclusion

The findings showed that the odds of CRC increased with greater food consumption with high GI and GL. Also, it was indicated that the CRC odds increases with an increase in IL. Further, this study found that individuals with the highest tertile of CQI and LCDS had the lowest CRC odds. Therefore, it is recommended to use a diet with high-quality carbohydrates and low GI and GL to minimize the odds of developing CRC. People should also be encouraged to have a balanced carbohydrate intake. Further studies are required to support these findings.

## Data Availability

The raw data supporting the conclusions of this article will be made available by the authors without undue reservation.
